# The E3 Ligase GmPUB21 Negatively Regulates Drought and Salinity Stress Response in Soybean

**DOI:** 10.3390/ijms23136893

**Published:** 2022-06-21

**Authors:** Yunhua Yang, Adhimoolam Karthikeyan, Jinlong Yin, Tongtong Jin, Rui Ren, Fei Fang, Han Cai, Mengzhuo Liu, Dagang Wang, Kai Li, Haijian Zhi

**Affiliations:** 1 National Center for Soybean Improvement, National Key Laboratory for Crop Genetics and Germplasm Enhancement, Key Laboratory of Biology and Genetic Improvement of Soybean—Ministry of Agriculture, Nanjing Agricultural University, Nanjing 210095, China; 2016201063@njau.edu.cn (Y.Y.); yinjinlong0000@126.com (J.Y.); 2018201084@njau.edu.cn (T.J.); fangfei1993@163.com (F.F.); cqch12345@163.com (H.C.); lmz@stu.njau.edu.cn (M.L.); smvwang@163.com (D.W.); 2 Subtropical Horticulture Research Institute, Jeju National University, Jeju 63243, Korea; karthik2373@gmail.com; 3 Center for Crop Genome Engineering, College of Agronomy, Henan Agricultural University, Zhengzhou 450046, China; renruinjau@163.com

**Keywords:** abscisic acid, abiotic stress, *GmPUB21*, ubiquitination, stomata

## Abstract

E3-ubiquitin ligases are known to confer abiotic stress responses in plants. In the present study, *GmPUB21*, a novel U-box E3-ubiquitin ligase-encoding gene, was isolated from soybean and functionally characterized. The expression of *GmPUB21*, which possesses E3-ubiquitin ligase activity, was found to be significantly up-regulated by drought, salinity, and ABA treatments. The fusion protein GmPUB21-GFP was localized in the cytoplasm, nucleus, and plasma membrane. Transgenic lines of the *Nicotiana benthamiana* over-expressing *GmPUB21* showed more sensitive to osmotic, salinity stress and ABA in seed germination and inhibited mannitol/NaCl-mediated stomatal closure. Moreover, higher reactive oxygen species accumulation was observed in *GmPUB21* overexpressing plants after drought and salinity treatment than in wild-type (WT) plants. Contrarily, silencing of *GmPUB21* in soybean plants significantly enhanced the tolerance to drought and salinity stresses. Collectively, our results revealed that *GmPUB21* negatively regulates the drought and salinity tolerance by increasing the stomatal density and aperture via the ABA signaling pathway. These findings improved our understanding of the role of *GmPUB21* under drought and salinity stresses in soybean.

## 1. Introduction

Plants are often subjected to abiotic stresses (i.e., drought, high temperature, salinity, flooding, and cold) that disrupt cellular metabolic balance and affect plant growth and development, resulting in reduced crop yield and quality [1]. Plants have evolved a variety of tolerance mechanisms including physiological, biochemical and molecular adaptations to these stress conditions. Drought and salinity stress are the two most common abiotic stresses, and plants respond to them by governing the expression of stress-responsive genes, hormones, and metabolites in their complex regulatory networks [2,3,4]. Thus, identifying and characterizing the major genes associated with stress responses is vital for understanding plant tolerance to stress. Using a combination of molecular and genetic approaches, several genes related to drought and salinity stress response have been discovered in many crops each with a particular role in tolerance or sensitivity [5,6,7].

Ubiquitination is a vital process found in eukaryotic cells, associated with diverse aspects of eukaryotic cellular regulation, in which ubiquitin molecules under the action of a series of special enzymes screen out intracellular target proteins and may change its localization, activity, and stability [8,9,10]. The ubiquitin-mediated post-translational modification of proteins plays a vital role in many cellular processes, including hormone regulation [11,12], environmental stress responses [13,14], DNA repair [15], cell cycle progression [16], organelle biogenesis, and self-incompatibility [17]. During this process, ubiquitination requires the step-wise transfer of ubiquitin to the target protein by three classes of the enzyme (E1, E2, and E3). Ubiquitin (Ub) is a 76-amino-acid protein found in all eukaryotic tissues. It is able to bind to Lys residues in the target substrate proteins [11,18]. The cascade begins with an E1 (or Ub-activating enzyme) catalyzing the formation of an acyl phosphoanhydride bond between the adenosine monophosphate (AMP) moiety of ATP and the C-terminal glycine carboxyl group of Ub, and then binding the Ub directly via a thiol-ester linkage between the Ub glycine and a cysteine in the E1. Then, by transesterification, E1 transfers the activated Ub to a ubiquitin-conjugating enzyme (E2). A ubiquitin ligase (E3) that can bind both the E2-Ub complex and the substrate aids in the establishment of an isopeptide bond between Ub and a substrate. E3 ligase specifies and distinguishes the target protein for degradation by the proteasome [19]. E3 ubiquitin ligases can be separated into diverse families according to domains and mechanism of action (i.e., HECT, RING, F-box, and U-box ligases) [20]. U-box is a modified RING-finger domain with 70 conserved amino acids. The plant U-box (PUB) protein is a well-known family of E3 ubiquitin ligases and plays a key role in plant response to abiotic stresses. Abscisic acid (ABA) is one of the important plant hormones and a key modulator that mediates the responses to abiotic stress [21,22]. ABA mediate stomatal closure, which reduce transpirational water loss, also plays a vital role in the activation of several downstream genes associated with the defense response. E3 ubiquitin ligases are one of the several gene families that respond to ABA. According to recent findings, PUB E3 ubiquitin ligases are thought to be negative regulators of ABA or ABA-associated responses [23,24].

The molecular mechanism of PUB genes from different plant species, such as Arabidopsis, rice, and soybean, have been identified and characterized. There are 64 PUB gene family members in Arabidopsis [25], 77 in rice [26], and 125 in soybean [27]. A few of these genes are involved in plant response to abiotic stresses and proclaimed the pathways. In Arabidopsis, four U-Box E3 Ubiquitin Ligases (*AtPUB18*, *AtPUB19*, *AtPUB22*, and *AtPUB23*) play negative roles in ABA-mediated drought response [24,28,29]. *AtPUB46* acts as a positive regulator in response to drought stress by reducing stomatal pore size and index in Arabidopsis [30]. In addition, *AtPUB11* was proposed as a negative regulator for drought tolerance by degrading the LRR1 and KIN7 RLKs [31]. *AtPUB30* plays a role in decreasing salinity tolerance by regulating the degradation of BKI1 [32,33]. *GmPUB6* and *GmPUB8* negatively regulate the drought tolerance and play an essential role in osmotic stress and ABA signaling pathway in soybean [27,34]. In rice, overexpressing *OsPUB15* exhibited tolerance to salinity stress than the wild type [35]. *OsPUB2* and *OsPUB3* act as positive regulators of the response to cold stress [36]. *OsPUB41* is a negative regulator in the dehydration conditions and interacts with *OsCLC6* [24]. *TaPUB1* and *TaPUB15* are positive regulators for salinity stress tolerance in wheat and rice [37,38]. In transgenic rice plants, *CaPUB1* shows a negative response for drought stress and a positive response for cold stress [39].

Soybean is an important oil crop around the world, and its production and quality are seriously constrained by salinity and drought stresses. In this study, we detailed the function of *GmPUB21* in terms of molecular biology and physiological indexes, found it increased stomatal density and negative responses to drought and salinity stress, moreover, *OE-GmPUB21* plants are more sensitive to ABA treatment. However, knock-down *GmPUB21* in soybean enhanced the tolerance to drought and salinity stress, which helped us to understand the functions of *GmPUB21* participating in drought and salinity stress response and the signal pathway.

## 2. Results

### 2.1. GmPUB21 Is a U-Box E3 Ligase in Soybean

**Figure 1:**
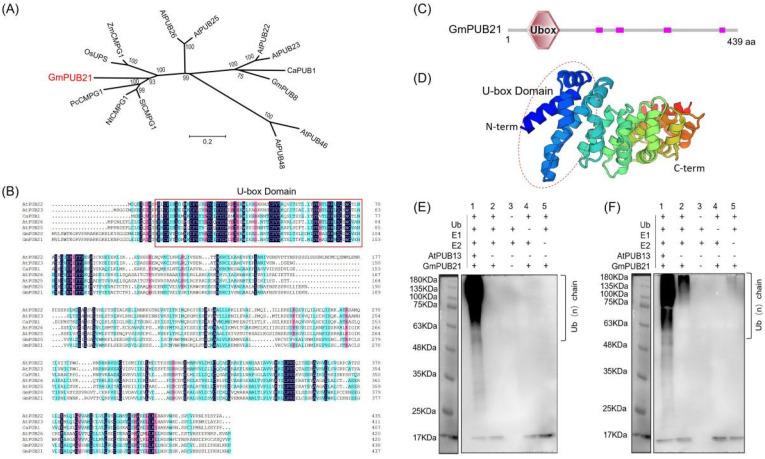
Cloning and characterization of the soybean *GmPUB21* gene encoding a E3 ubiquitin ligase. (**A**) Phylogenetic analysis of U−box type ubiquitin ligase (PUB) genes from soybean, Arabidopsis, parsley, rice, tobacco, tomato, and hot pepper. The dendrogram was conducted in MEGA4.0 software with the neighbor−joining method. (**B**) Multiple sequence alignment of amino acid sequences of GmPUB21 and other U−box type ubiquitin ligase (PUB) genes with DNAMAN. (**C**) Schematic structures of GmPUB21, open bars indicate coding regions, hexagon represent the U−box motif, and pink bars depict the low complexity. (**D**) The structure of GmPUB21 peptides was constructed by homology modeling using SWISS−MODEL. (**E**) The purified His−GmPUB21 was used for self−ubiquitination assays. (**F**) The pre−incubated His−GmPUB21 was used for self-ubiquitination assays.

In soybean, we identified a gene, *Glyma.02G242900* (designated as *GmPUB21*), containing a U-box domain. *GmPUB21* belongs to a family member of the U-box E3-ubiquitin ligase that is classified as a GKL-type (conserved Glycine (G), Lysine (K)/Arginine (R) residues and its leucine-rich feature) and positioned on chromosome 2. To know the evolutionary relationship among GmPUB21 and other reported PUB proteins, amino acid sequences of 16 PUB genes were used to perform phylogenetic analysis, and the results show that GmPUB21 is relatively close to those proteins up to 68.2%, especially to NtCMPG1 and PcCMPG1 (Figure 1A). Multiple sequence alignment revealed that the U-box domain of GmPUB21 is conserved with other PUB proteins (Figure 1B).

The total length of GmPUB21 is 1317 bp without exons and the U-box domain at its N-terminal, encoding amino acids of 439 aa residues with a calculated molecular mass of 48.5 kDa and a theoretical pI of 8.27 (Figure 1C,D). U-box proteins are known to have E3 ubiquitin ligase activity. To examine whether GmPUB21 is a functional E3 Ub ligase, in vitro self-ubiquitination assay experiment on bacterially expressed His-GmPUB21. The purified His-GmPUB21 did not show E3 ligase activity during the existence of human E1 (UBE1), human E2 (UBCh5c), and His-tagged ubiquitin, although the reactions are deficient in E1, E2, or ubiquitin. However, AtPUB13 (Positive control) exhibited a higher self-ubiquitination activity (Figure 1E), and it was confirmed by detecting the high-molecular-weight bands with an antibody to ubiquitin, which means that the proteins have been labeled by ubiquitin molecules. Post-translational modification is also crucial for E3 enzyme activity, so we speculate that the premise of GmPUB21 enzyme activity may also require post-translational modification. Therefore, purified His-GmPUB21 was pre-incubated with soybean leaf crude extract before using it in the in vitro ubiquitination experiment. It revealed that pre-incubated His-GmPUB21 possesses E3 ligase activity when E1, E2, and ubiquitin were present. However, no protein ubiquitination was seen in the absence of E1, E2, or ubiquitin (Figure 1F). These results reveal that GmPUB21 requires post-translational modification to exhibit E3 ligase activity.

### 2.2. GmPUB21 Responses to Osmotic Stress and ABA Treatment, Localize to the Nucleus, Cytosol, and Plasma Membrane

**Figure 2:**
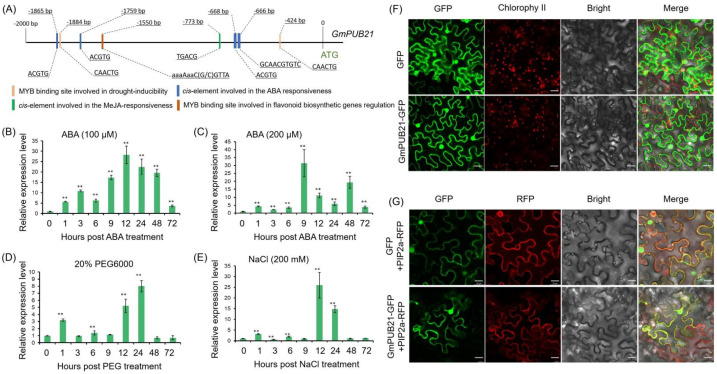
The expression pattern *GmPUB21* in soybean under different treatments and subcellular localization. (**A**) Predicted *cis*−acting elements in the promoter sequence (~2000 bp) of *GmPUB21* with PlantCARE. [(**B**) ABA (100 µM), (**C**) ABA (200 µM), (**D**) 20% PEG, and (**E**) NaCl (200 mM)] were analyzed using quantitative real-time PCR analysis. Leaves of soybean from all the treatments collected at 0, 1, 3, 6, 9, 12, 24, 48, and 72 h after treatment were used for the analysis. *Tubulin* was used as the internal control. Data are means ± SD from three biological replicates. Asterisks indicate statistically significant differences relative to water treatment (Student’s *t*-tests, ** *p* < 0.01). (**F**) Subcellular localization of GmPUB21 in *N. benthamiana*, GmPUB21−GFP fusion protein or GFP alone expressed in tobacco leaves. (**G**) Co−localization of GFP fusion proteins with plasma membrane marker (PIP2a) in tobacco leaves. The fluorescence signals were monitored by confocal microscopy, and the images were taken at 2 days post−infiltration. Scale bars, 20 μm.

To analyze the biological functions that *GmPUB21* may be involved in, the 2000-bp upstream promoter sequence of *GmPUB21* was analyzed, and it was found that there are *cis*-elements involved in the ABA responsiveness and MYB binding sites involved in drought-inducibility in the promoter region (Figure 2A). Hence, we speculated that *GmPUB21* may be involved in drought stress response and ABA signal response. To examine the expression pattern of *GmPUB21*, qRT-PCR was used to detect the expression of *GmPUB21* in soybean seedlings that were subjected to 20% PEG6000, salinity (200 mM NaCl), and ABA (100 µM and 200 µM). A significant up-regulation was found at 1 h on *GmPUB21* after PEG6000 treatment, and the highest expression level was detected at 24 h. However, the expression of *GmPUB21* was decreased when the treatment was prolonged to 48 h (Figure 2D). The expression pattern of *GmPUB21* to salinity stress was similar to the PEG6000 treatment. *GmPUB21* expression reached a high level at 12 h and then decreased at 48 h (Figure 2E). There was continuous up-regulation observed on *GmPUB21* following ABA treatment. In particular, the expression level of *GmPUB21* was high from 9 h to 48 h (Figure 2B,C). Collectively, these results suggest that the *GmPUB21* may respond to Osmotic stress and ABA signal. To investigate the subcellular localization of the GmPUB21, the *GFP* alone (the control) and *GmPUB21-GFP* construct were transiently expressed in *Nicotiana benthamiana* (*N*. *benthamiana*) leaves using agroinfiltration. The GmPUB21-GFP showed a strong fluorescence signal and was uniformly distributed throughout the cytosolic fraction and nuclei of cells, which is similar to GFP alone (Figure 2F). To further analyze the localization of GmPUB21, plasma membrane (*PIP2a-RFP*) was co-expressed in *N. benthamiana* with *GmPUB21-GFP*. The results showed that *GmPUB21-GFP* could overlap with the plasma membrane marker PIP2a-RFP (Figure 2G). The results revealed that GmPUB21 is localized in the cytoplasm, nucleus, and plasma membrane.

### 2.3. Overexpression of GmPUB21 Inhibits the Seed Germination under Mannitol, NaCl, and ABA Treatment

**Figure 3:**
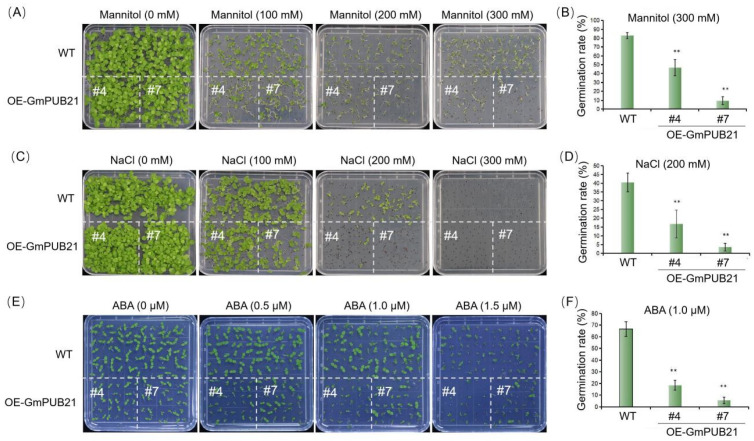
Seed germination of transgenic (*GmPUB21* overexpressing tobacco plants, *OE-GmPUB21#4* and *OE-GmPUB21#7*) and wild type (WT) tobacco plants under control and different treatments. Seeds from transgenic and WT plants were grown on the Murashige–Skoog (MS) medium with different concentrations of (**A**) Mannitol (0–300 mM), (**C**) NaCl (0–300 mM) for 12 days and (**E**) ABA (0–1.5 µM) for 8 days. The germination rate of seeds treated with (**B**) 300 mM mannitol, (**D**) 200 mM NaCl for 12 days, and (**F**) 1.0 µM ABA for 8 days. For each replicate, more than 30 seeds were measured. The data shown are the means ± SD of three independent experiments. Asterisks indicate statistically significant differences relative to WT (Student’s *t*-tests, ** *p* < 0.01).

To better understand the role of *GmPUB21* under abiotic stresses, we adopted an overexpression strategy. Transgenic tobacco plants were produced by over-expressing the *GmPUB21* under the control of the CaMV 35S promoter. Six transgenic tobacco lines were produced, and two T_2_ homozygous lines (*OE-GmPUB21#4* and *OE-GmPUB21#7*) with the highest expression of *GmPUB21* were selected and harvested seeds. Those lines of T_3_ seeds were used for further experiments (Figure S1). The germination percentage of transgenic T_3_ homozygous lines (*OE-GmPUB21#4* and *OE-GmPUB21#7*) and wild-type (WT) plants under osmotic stress (mannitol and NaCl) and ABA treatment. Under normal conditions, there were no noticeable changes in the germination among the transgenic and WT plants (Figure 3A,C,E), while the germination was inhibited after treatment with mannitol, NaCl (salinity) for 12 days, and ABA for 8 days at different concentrations. The inhibition was more remarkable for transgenic seeds. For instance, more than 80% of WT seedlings grown in the presence of 300 mM mannitol had reached the radicle elongation stage and open cotyledons. However, transgenic seedlings just accounted for 50% of the total (Figure 3B). A similar response was observed in NaCl and ABA treatments. In response to 200 mM NaCl, more than 40% of WT plants attained the radicle elongation stage and opened the cotyledons, but the proportion of transgenic seedlings was <17% (Figure 3D). More than 60% of WT plants attained the cotyledons open stage at 1.0 µM ABA, but the proportion of transgenic seedlings was <20% (Figure 3F). The results suggest that *GmPUB21* OE-plants are less tolerant to mannitol, salinity, and ABA treatments at the seed germination stage.

### 2.4. Overexpression of GmPUB21 Increases the Sensitivity to Drought and Salinity Stress

**Figure 4:**
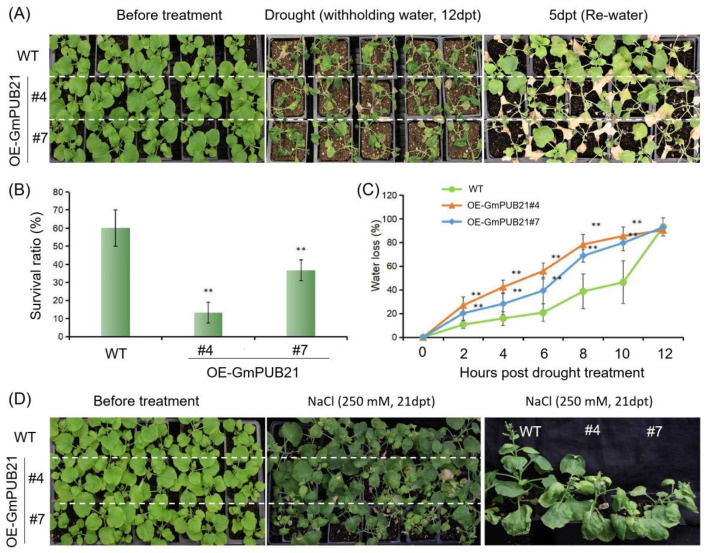
Overexpression of *GmPUB21* tobacco plants is sensitive to drought and salinity stress. (**A**) 3-week-old seedlings were grown under normal conditions when exposed to drought stress by withdrawing the water for 12 days, and recovered for 5 days. (**B**) The survival rate was calculated as the percentage of plants that resumed growth 5 days after rewatering and survival rate values represent means ± SD (*n* = 30). (**C**) Comparison of the water loss from detached rosette leaves of the WT and *35S:GmPUB21* plants at the time points indicated. Water loss was expressed as a percentage of the initial fresh weight. Data shown are the means ± SD of three independent experiments. Asterisks indicate statistically significant differences relative to WT (Student’s *t*-tests, ** *p* < 0.01). (**D**) 3-week-old seedlings of transgenic and WT plants undergo salinity stress (250 mM NaCl for 21 days) and the phenotype differences were observed in transgenic and wild-type plants after salt treatment by 250 mM NaCl for 21 days, dpt = days for post treatment. The experiments were repeated three times and similar results were obtained.

We further examined the transgenic and WT tobacco plant responses under drought and salinity stresses. For drought response, 3-week-old *OE-GmPUB21* plants and WT plants were withdrawing the water for 12 days. Then, we re-watered the plants and calculated the survival rate between transgenic and WT plants (Figure 4A). It showed that WT plants (60%) had a better recovery than transgenic plants (<37%) (Figure 4B). Moreover, we measured the water loss rate of leaves in transgenic and WT plants. With the time extension, the water loss rate gradually increased. After leaves were detached for 10 h, two transgenic lines lost 85.5% and 79.7% of their initial fresh weight, respectively, compared to 46.3% in WT (Figure 4C), indicating that water loss of detached leaves in transgenic plants was much faster than WT. On the other hand, 3-week-old transgenic and WT plants were treated with 250 mM NaCl to analyze the salinity stress response. When subjected to salinity stress, the WT plants did not show any difference and grew normally, while the transgenic plants were dwarfed, and the apical growth was inhibited. The middle and upper leaves of WT plants are healthy and flat; conversely, transgenic plants became curved outward, thicker, and uneven (Figure 4D).

**Figure 5:**
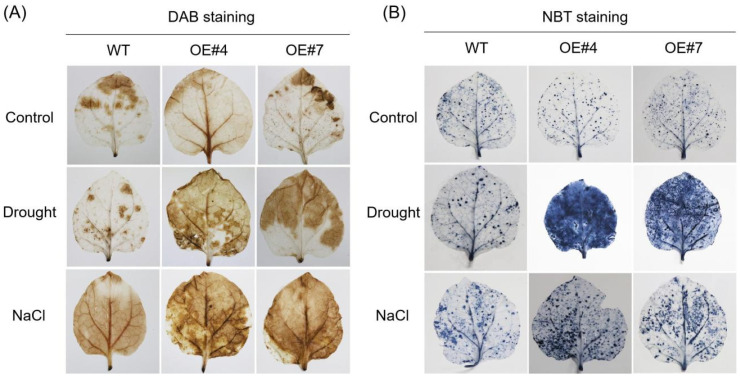
Overexpression *GmPUB21* increases ROS accumulation under drought and salt stress. (**A**) Histochemical analysis of H_2_O_2_ by DAB staining and (**B**) analysis of O_2_^−^ by NBT staining. The 4-week-old transgenic and WT tobacco plants were subjected to drought stress or salt stress (250 mM NaCl) for 3 days and then leaves were collected and analysed for ROS accumulation. The experiments were repeated three times and similar results were obtained.

The reactive oxygen species (ROS) accumulation causes damage to different cellular components and macromolecules (i.e., plasma membrane, nucleic acids, and proteins) and results in cell death. Therefore, we investigated the H_2_O_2_ and O_2_^−^ generation in transgenic and WT tobacco plants. For this, 4-week-old plants were exposed to drought and salinity stress for 3 days. Histochemical staining revealed that more H_2_O_2_ and O_2_^−^ accumulated in transgenic plants than in WT plants under drought and salinity stress; however, the content of H_2_O_2_ was no significant difference between *OE-GmPUB21* and WT plants under normal conditions. A similar result was also found in the O_2_^−^ (Figure 5A,B). This shows that transgenic plants produce more ROS during drought and salinity stress than WT plants. Taken together, the results suggest that overexpression of *GmPUB21* in tobacco resulted in more sensitivity to salinity and drought stress.

### 2.5. The Stable Genetic Transformation of GmPUB21 Promoted Its Expression in Guard Cells and Increased Stomatal Density

**Figure 6:**
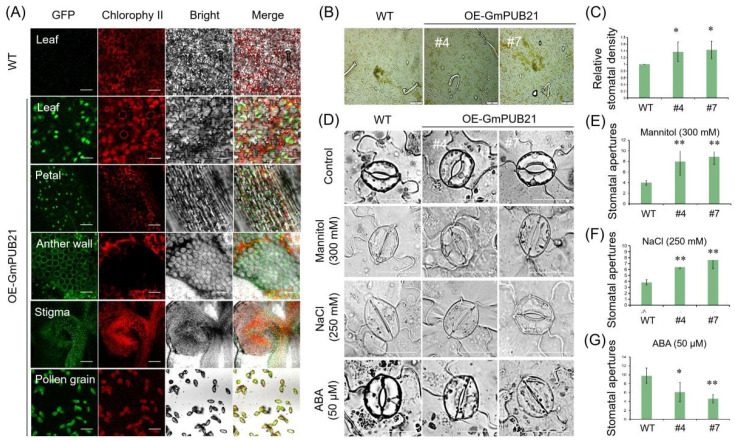
The GmPUB21 effect in the stomatal density and aperture. (**A**) Image of GmPUB21-GFP in transgenic plants at different tissues. Scale bars, 50 μm. (**B**) Image of stomata of transgenic and wild type plants under bright-field microscopy. (**C**) Statistical analysis of stomatal density. The stomatal of WT as standard “1”. Data shown are the means ± SD of three independent experiments. Asterisks indicate statistically significant differences relative to WT (Student’s *t*-tests, *n* = 4 plants, * *p* < 0.05). Scale bars, 20 μm. (**D**) Image of stomata aperture in transgenic and wild type plants under normal condition and drought (300 mM Mannitol) for 3 h, salinity (250 mM NaCl) for 3 h, ABA (50 μM) for 2 h, the average size of stomatal apertures for each genotype, Scale bars, 10 μm. (**E**–**G**) Stomatal apertures measured under 300 mM Mannitol, 250 mM NaCl, and 50 μM ABA from three replicates, with at least 50 stomatal apertures in epidermal peels measured per replicate. Data shown are means ± SD of three independent experiments. Asterisks indicate statistically significant differences relative to WT (Student’s *t*-tests, *n* > 50, * *p* < 0.05, ** *p* < 0.01).

*OE-GmPUB21* plants were sensitive to drought and salinity stress, leading us to wonder what the difference between transgenic plants and wild-type plants is. Interestingly, when we observed the fluorescence in transgenic plants (*35S: GmPUB21:GFP*) and found that the GFP Fluorescence signal was evenly distributed in different tissues, but the signal is stronger in the nucleus of leaf guard cells than other tissues (Figure 6A). We speculate that there is an important relationship between *GmPUB21* and stomata. First, we examined the differences in stomata between the transgenic and WT tobacco plants. It revealed that the stomatal density of transgenic plants was higher than WT (Figure 6B), and the stomata of the transgenic plants increased approximately 20–30% more than WT plants (Figure 6C). It suggested that *GmPUB21* regulation of drought and salinity tolerance may be linked to the physiological process of guard cells. We further analyzed the stomatal closure between transgenic and WT plants under drought, salinity, and ABA treatment. It revealed that under the normal conditions, stomatal apertures were no difference between OE-GmPUB21 and WT plants. However, under 300 mM mannitol and 250 mM NaCl treatment, the average stomatal apertures in WT plants were 3.84 and 4.02 μm, respectively, and it was significantly lower than in transgenic plants [*OE-GmPUB21#4* (7.15 and 7.98 μm) and *OE-GmPUB21#7* (7.87 and 8.9 μm)]. The stomata in many WT-plants were closed, while they remained open in many transgenic plants (Figure 6D–F). On the contrary, under ABA treatment (50 µM), the stomatal aperture of WT plants (9.79 μm) was higher than transgenic plants (6.14 μm and 4.59 μm). The stomata of WT remained open, while the stomatal opening of transgenic lines decreased, especially for *OE-GmPUB21#7*, the stomata were almost entirely closed (Figure 6D,G). Collectively, the results revealed that *GmPUB21* might play a crucial role in stomatal development and ABA-mediated stomatal closure.

### 2.6. Knock-Down GmPUB21 in Soybean Improves the Tolerance to Drought and Salinity Stress

**Figure 7:**
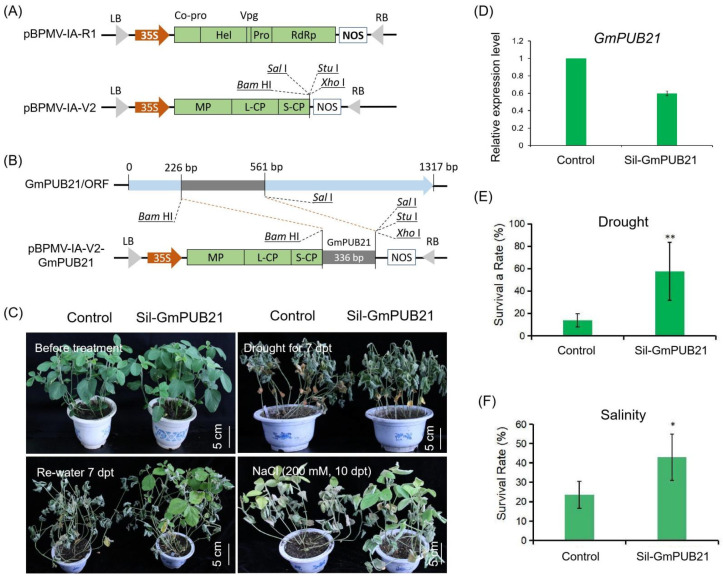
*Knock-down GmPUB21* enhanced the tolerance to drought and salinity stress in soybean. (**A**) BPMV genomic RNA1 and RNA2 are expressed under the control of the CaMV 35S promoter and Nos terminator. (**B**) Schematic diagram and related information of gene-silencing vectors for GmPUB21. (**C**) Phenotypes of control (V) and *SilGmPUB21* under drought (water withholding for 7 days) and rewater for 7 days, salinity stress (200 mM NaCl) for 10 days, respectively. (**D**) The *GmPUB21* relative expression in control (V) and *SilGmPUB21 plants.* (**E**) The survival rate of control (V) and *SilGmPUB21* after re-watering 7 days. (**F**) The survival rate of control (V) and *SilGmPUB21* after treatment with 200 mM NaCl for 10 days. dpt = days for post-treatment. The experiments were repeated three times and similar results were obtained (Student’s *t*-tests, *n* = 45, * *p* < 0.05, ** *p* < 0.01).

To further confirmed the function of the *GmPUB21* in soybean, we used the BPMV-based gene construct to knock down the *GmPUB21* and introduced it into soybean cv Williams 82 along with control BPMV empty vector (V) (Figure 7A,B), as well as analyze the gene expression of *GmPUB21* using PCR and qRT-PCR analyses (Figure 7D and Figure S3). This revealed that *GmPUB21* expression in the *Sil-GmPUB21* plants were decreased to 60% compared to the control (V) plants. The *Sil-GmPUB21* plants had drought stress imposed by withdrawing the water. All plants showed dehydration, wilting, and withered, exhibiting more severe symptoms than control (V) plants after drought stress for 7 days. Then, plants were re-watering for 7 days, and *Sil-GmPUB21* plants recoved better than control (V) plants (Figure 7C); after re-watering for 7 days, the survival rates of *Sil-GmPUB21* plants were higher (57.4%) compared to 13.9% for control (V) plants (Figure 7E). Further, to determine the *Sil-GmPUB21* plants’ response to salinity stress, 200 mM NaCl was applied to *Sil-GmPUB21* and control (V) plants. After 10 days of treatment, plants showed yellowing and withering (Figure 7C). The survival rates of *Sil-GmPUB21* plants were 42.94%, higher than the control (V) plants, which were 23.5% (Figure 7F). Collectively, these results suggest that knock-down *GmPUB21* improved the soybean tolerance to drought and salinity stress.

## 3. Discussion

The plant U-box (PUB) gene is one of the largest families found in plants, and they play diverse roles in plant development, biotic and abiotic stress responses [30,40]. In the recent past, genes belonging to the PUB gene families have been discovered and functionally described in many crops, including *AtPUB11* in Arabidopsis [31], *OsPUB2*, *OsPUB3*, and *OsPUB41* in rice [24,36], *TaPUB1* in wheat [37], and *GmPUB8* in soybean [27]. The available studies suggest that the PUB genes are important regulators of abiotic stresses [29,30,34,38]. The present study identified and functionally characterized the U-box E3 ubiquitin ligase gene *GmPUB21*, which was significantly induced by drought, salt, and ABA treatments and localized in the cytoplasm, nucleus, and membrane, these results are consistent with the OsPUB67 reported by Qin et al. [41]. Moreover, PlantCARE predicted the upstream sequence of *GmPUB21* and reveals it containing MYB binding site involved in drought-inducibility and flavonoid biosynthetic, cis-element involved in the ABA and MeJA-responsiveness. We speculate that *GmPUB21* may revolve in a wide range of biological processes.

Ubiquitination is an important post-translational modification in eukaryotes [42]. It has been demonstrated that specific E2 Ub-conjugating enzymes play important role in ubiquitination. *AtUBC8* or *AtUBC9*, an E2 enzyme from Arabidopsis, showed better activity than *AtUBC7* in detecting polyubiquitination of the E3 ligase *OsPUB73* [7]. It has been reported that *OsPUB67* strongly interacted with six E2s. However, the in vitro ubiquitination assay revealed that *OsPUB67* exhibited polyubiquitination only with *OsUBC18* or *OsUBC23* [41]. In addition, post-translational modification also plays a vital role in the E3 ligase activity. For instance, the E3 ligase activity of *OsPUB15* depends on pre-phosphorylated by *PID2K* [43]. In our study, a pre-incubation crude protein strategy was applied to detect the ubiquitination activity of *GmPUB21*. It detected the polyubiquitination signal and confirmed that *GmPUB21* had E3 ligase activity, it shows post-translational modification is vital for the E3 ubiquitin activity. However, additional research will help to explore in more detail about *GmPUB21* self-ubiquitin activity.

As reported in previous studies, overexpressing PUB genes negatively regulate the drought and salt stress response in some plant species, such as *PUB11*, *PUB22*, *PUB23,* and *AtPUB30* in Arabidopsis [31,32,40], *OsPUB41* and *OsPUB75* in rice [24,44], *GmPUB6* and *GmPUB8* in soybean [27,34], and *TaPUB26* in wheat [45]. In our study, overexpressing *GmPUB21* shows sensitive to drought, salinity stress and ABA treatment (Figure 3), and more ROS (O_2_^−^ and H_2_O_2_) accumulated in transgenic plants after drought and salinity treatment, which may disturb the tolerance to drought and salt stress. However, the opposite phenotype was obtained in *GmPUB21* down expression plants. Similar results were obtained by Yan et al. [46] and Ren et al. [47].

Water-generated turgor pressure is also a driving force of cell expansion. Plant cells usually adjust their osmotic potential to meet the requirement of the whole plant in balancing its water budget. Significant changes in water potentials in the environment can impose osmotic stress on plants, especially for when high salinity and drought are the major factors [48], which disrupts normal cellular activities: the consequences of osmotic stress manifest in the inhibition of cell elongation, stomata closure, reduction of photosynthetic activity, disturbances in water and ion uptake, translocation of assimilates, and changes of various metabolic processes or even causing plant death [49]. PEG, mannitol or sorbitol, and NaCl are widely applied to stimulate osmotic stress [50,51,52], and it was found that by reducing the same osmotic potential, the concentration is different for various osmolytes. For example, 150 mM NaCl corresponds to 300 mM mannitol in osmotic pressure, as similarly found by [49,53]. In this study, we studied the OE-GmPUB21 plants respond to osmotic stress with mannitol and NaCl, the results showed that the germination rate of tobacco seeds were inhibited both by mannitol and NaCl treatment. However, the effect of NaCl on seed germination was more obvious than mannitol at the same concentration, and the germination rate at 100 mM NaCl was close to 200 mM mannitol. Similarly, seeds germination at 200 mM NaCl was close to 300 mM mannitol. Those results were consistent with the previous research results.

Stomata play a vital function in controlling the gas exchange and regulating transpiration rate. In recent years, many studies detailed the stomata’s role in abiotic stress tolerance. When *GmPUB21-GFP* transiently expressed in tobacco leaves, the green fluorescence signal could be evenly expressed in epidermal cells, but not in guard cells. The control (GFP alone) also found that it could not be expressed in guard cells (Figure 2F,G). However, when *GmPUB21-GFP* was inserted into plant genomics, the fluorescence signal was found in different tissues, especially in guard cells (Figure 6A). This suggested that GmPUB21 mainly accumulates in guard cells. Adler et al. [54] reported that overexpressing *AtPUB46* improved the tolerance to drought and oxidative stress and reduced the leaf blade width, while the leaf length, number of leaves in the rosette, the stomatal density, and water loss rates were not statistically different between *AtPUB46-OE* and WT plants. In another study, overexpression of the histone deacetylase HDA704 in transgenic rice enhanced the drought and salt tolerance by promoting stomatal closure, reducing the number of stomata, and slowing the rate of water loss [55]. Instead, Pu-miR172d was expressed in the guard cells of young leaves and the stomatal density was significantly reduced, while the water-use efficiency and drought tolerance were increased in Pu-miR172d-overexpressed plants by decreasing the net photosynthetic rate, stomatal conductance, and transpiration [56]. In our study, there was a difference in stomatal density. Transgenic tobacco plants of GmPUB21 significantly improved the stomatal density and water-loss rate compared to WT plants (Figure 4B,C). Similarly, under osmotic conditions, the stomatal aperture of *OE-GmPUB21* was higher than WT plants. Thus, we speculate that the impact of overexpressing *GmPUB21* on drought and salt tolerance is primarily from the increasing stomatal density and stomatal aperture, these two factors lead to increased water loss, which reduces the resistance of plants to drought and salinity.

ABA is an important phytohormone that is reported to be involved in the stress response, promoting embryo maturation and maintaining seed dormancy while inhibiting seed germination, root growth, and stomatal closure [57,58]. In addition, the ABA-responsive genes may strengthen or weaken the plants’ tolerance to abiotic stresses [59,60]. Down-regulation of *AtPUB19 resulted in* *hypersensitivity* to ABA, and increased the ABA-induced stomatal closing and drought tolerance; this means *AtPUB19* is a negative regulator of ABA signaling [29]. *TaFBA1* OE plants show tolerance to drought and ABA, stomatal closure of OEs plants was slower than WT after ABA treatment, suggesting *TaFBA1* positively regulates plant drought tolerance but negatively regulates the ABA signaling pathway [61]. *GmPUB6* and *GmPUB8* were hypersensitive to drought but inhibit the stomatal closure under the drought and ABA; therefore, they are all as negative regulators of ABA [27,34]. In our study, *GmPUB21* expression was up-regulated by ABA. However, the transgenic plant’s seed germination was significantly inhibited under ABA treatment. However, the stomatal closure of transgenic plants was faster than WT plants after ABA treatment, and slower after drought and salt treatments. These results demonstrated that *GmPUB21* plays a positive regulatory role in the ABA signaling pathway, but negatively regulates salinity and drought stress.

Protein ubiquitination is a post-translational modification that alters the surface of substrate proteins, possibly affecting properties such as stability and activity, as well as driving protein interactions and subcellular localization [8,62]. *PalWRKY77* is a negative transcriptional regulator of ABA signaling, decreasing the tolerance to salt stress and inhibiting the transcription factors of *PalNAC002* and *PalRD26* expression in poplars [63]. PalPUB79 enhanced drought tolerance and positively regulates hypersensitivity to ABA in transgenic poplars. PalPUB79 interacted with *PalWRKY77* and mediated its ubiquitination for degradation, therefore counteracting its inhibitory effect on *PalRD26* transcription [33]. These results show that the E3 ligase has biological functions diametrically opposed to those of its substrate, which is recognized explicitly and degraded by the 26S proteasome. These results imply that *GmPUB21* may target its substrate protein and impair the tolerance to drought and salinity.

**Figure 8:**
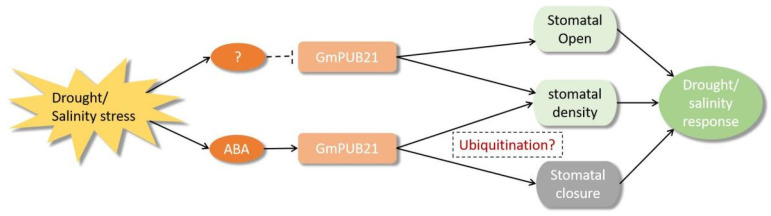
Modes of action of the *GmPUB21* U-box E3 ligase was divided into two different pathways: ABA-dependent and other signals. *GmPUB21* as a positive effect on the ABA signaling pathway promote the stomatal closure. The other signal by which GmPUB21 regulates drought and salinity responses is not known. Overexpressing *GmPUB21* leads to increasing the stomatal density, and also regulated the stomatal aperture in response to drought and salinity stress. In addition, *GmPUB21* as a U-box E3 ligase may involved in the ubiquitination pathway to regulate the development of stomata and the response to drought and salt stress.

In summary, we indicated that *GmPUB21* played a negative role in drought and salinity stress (Figure 8). *GmPUB21* possessing E3 liagase activity was regulated by drought, salinity, and ABA. Overexpression lines of *GmPUB21* have a lower germination under mannitol, NaCl and ABA. At the same time, lower survival rate and higher leaf water-loss rate under drought treatment was found. Moreover, stomatal density was improved in *OE-GmPUB21* plants. The stomatal aperture of *OE-GmPUB21* was higher under mannitol and NaCl, while it nearly closed after ABA treatment compared with the WT plant. Consistently, knock-down *GmPUB21* in soybean plant increased the drought and salinity tolerance, indicating that *GmPUB21* is involved in drought and salinity stress partially by regulating the stomatal development and movement. In addition, *GmPUB21* plays a crucial role in drought and salinity stress through an ABA-dependent pathway. However, more details have to be investigated in order to know other components that interact with GmPUB21 or find out the substrate that is regulated by the 26S proteasome to obtain a better understanding of the molecular mechanisms underlying *GmPUB21* under abiotic stress.

## 4. Materials and Methods

### 4.1. Plant Materials and Treatments

Soybean cultivars Williams 82, Nannong 1138-2, and tobacco (*Nicotiana* *benthamiana)* seeds were provided by National Center for Soybean Improvement, Nanjing Agricultural University, Nanjing, China, and were used in this study. We used the soybean cv. ‘Williams 82’ to isolate the *GmPUB21* gene sequence and analysis gene expression. Williams 82 seeds were sown in pots (A190, outside diameter 16.5 cm, inside diameter 13.5 cm, height 11 cm, bottom diameter 9 cm) with the ratio 2:1 of vermiculite:nutrient soil. Seedlings were grown in the greenhouse at 25 ± 2 °C under photoperiod conditions (16 h light/8 h dark) with a relative humidity of 60 ± 5% and artificial light (LED) was used to provide the light source with the illumination of (100 ± 5 µM photons/s/m^2^). Also, 10-day soybean seedlings were subjected to 20% PEG6000, salinity (200 mM NaCl), and ABA (100 µM and 200 µM) treatments. There were 8-10 soybean seedlings in each pot (A190), for PEG and NaCl treatment, each pot was irrigated with 200 mL 20% PEG6000 and 200 mM NaCl; for ABA treatment, 100 µM and 200× µM solution of ABA was sprayed on leaf surface, each pot evenly sprayed 5 mL ABA solution. After treatment, leaves from all the treatments were collected at 0, 1, 3, 6, 9, 12, 24, 48, and 72 h, then immediately frozen into liquid nitrogen and stored at −80 °C.

### 4.2. Cloning and Sequence Analysis of GmPUB21

Total RNA was isolated from leaves of soybean cultivar ‘Williams 82’ using Trizon Reagent (CW0580S, CWBIO, Beijing, China), following the user guidelines. First-strand complementary DNA (cDNA) synthesis was done using PrimeScript™ II 1st Strand cDNA Synthesis Kit (6210A, TaKaRa, Dalian, China) following the user’s manual. The complete cDNA sequence of *GmPUB21* was amplified by PCR with Prime STAR Max DNA Polymerase (R045A, TaKaRa, Dalian, China) and primers described in Table S1. The gene model found in the *Glyma 2.0* assembly on https://www.soybase.org (accessed on 5 June 2018). was used to design primers for *GmPUB21* (*Glyma.02g242900*). The amplified products were purified and cloned into T-Vector pMD^TM^20 (3270, TaKaRa, Dalian, China) and confirmed by sequencing (Invitrogen, Biotechnology Co., Ltd., Shanghai, China). Promoter element prediction and analysis using plantCARE (http://bioinformatics.psb.ugent.be/webtools/plantcare/html/) (accessed on 20 August 2018). Protein domains of the obtained sequences were analyzed using SMART (http://smart.embl-heidelberg.de/) (accessed on 1 January 2018). The structure of peptides constructed by homology modeling using SWISS-MODEL (https://swissmodel.expasy.org/interactive) (accessed on 20 April 2020). The homologous sequences were searched using the BLASTP program at GenBank (http://www.ncbi.nlm.nih.gov/blast) (accessed on 1 January 2018) in the Non-Redundant (NR) database. Multiple sequence alignment was done by DNAMAN. The phylogenetic analysis was carried out using PUB genes from other plants, including Arabidopsis, tomato (*Lycopersicon esculentum*), banana (*Musa acuminata*), maize (*Zea mays*), Parsley (*Petroselinum crispum*), rice (*Oryza sativa*), tobacco (*Nicotiana benthamiana*), soybean (*Glycine max*), and pepper (*Capsicum annuum*). The sequences were obtained from Phytozome.13 (https://phytozome-next.jgi.doe.gov/) (accessed on 19 May 2022) and Sol Genomics Network (https://solgenomics.net/organism/Nicotiana_benthamiana/genome) (accessed on 12 January 2021), including AtPUB22 (AT3G52450), AtPUB23 (AT2G35930), AtPUB26 (AT1G49780), AtPUB25 (AT3G19380), AtPUB46 (At5G18320), AtPUB48 (At5G18340), CaPUB1 (Capang08g000552), GmPUB8 (Glyma.02G195900), GmPUB20 (Glyma.14G212200), GmPUB21 (Glyma.02G242900), OsUPS (LOC_Os03g13740.1), SlCMPG1 (Solyc01g005160.4.1), ZmCMPG1 (Zosma01g30390), TaPUB1 (Traes_4DL_99308AE34.1), NtCMPG1(Niben101Scf00984g09007.1), and PcCMPG1 (AAK69402). The phylogenetic tree was built using the neighbor-joining technique (NJ) with 1000 bootstraps in MEGA version 5.

### 4.3. Quantitative Real-Time PCR Analysis

Trizol Reagent (CW0580S, CWBIO, Beijing, China) was used to extract the total RNA, and first-strand complementary DNA (cDNA) was synthesized using HiScript^®^ III-RT SuperMix for qPCR (+gDNA wiper) (R223-01, Vazyme, Nanjing, China). The quantitative real-time PCR mixture contained 2 μL of cDNA, 0.8 μL of forward primer and 0.8 μL of reverse primer, 10 μL of 2× ChamQ Universal SYBR qPCR Master Mix (Q311-01, Vazyme, Nanjing, China), 6.4 μL of dd H_2_O, totally in a 20 μL final volume. All the reactions were done in three replications in 96-well plates using the Roche Real-Time PCR system (LightCycler480 II, Roche) following thermal conditions: 95 °C for 30 s, 95 °C for 5 s, and 57 °C for 45 s for 40 cycles. To normalize the sample variance, the soybean β-*Tubulin* gene (NM_001252709.2) was used as the internal control. The relative expression was measured through comparison with that of the normal condition plants (set as 1.0) and analyzed using the 2^−^^△△Ct^ method [64]. The gene-specific primers used for quantitative real-time PCR analysis are listed in Table S1.

### 4.4. Recombinant Protein Expression and Purification

The full-length *GmPUB21* coding sequence was cloned into *pET32a* (His tag) to generate *His-GmPUB21*, and the positive clones were selected and sequenced (Invitrogen, Biotechnology Co., Ltd., Shanghai, China). Recombinant *His-GmPUB21* was expressed in *Escherichia coli* strain BL21 (DE3) and purified by Ni-NTA resin. Briefly, overnight bacteria cultures were diluted 100 times in a total volume of 100 mL Luria–Bertani medium and incubated at 37 °C until an OD600 of 0.5 was achieved. The His-GmPUB21 proteins were induced for 3 h at 30 °C with 0.5 mM Isopropyl-D-Thiogalactoside (IPTG). Bacteria were extracted after induction by centrifugation at 12,000 rpm for 10 min and suspended in 1×PBS (Tris-HCl pH 8.0, 50 mM NaCl, 0.1% TritonX-100) [65]. The suspension was then sonicated (FB705220 Fisher 705, Waltham, MA, USA) and centrifuged. The resultant supernatant was filtered by affinity chromatography via HisPur Ni-NTA Resin (88831, Thermo Scientific™, Shanghai, China) following the user guidelines. The primers used in this assay are listed in Table S1.

Coomassie Blue Staining revealed that His-GmPUB21 has not been purified from the supernatant, and the protein mainly exists in the precipitation (Figure S2A). His-GmPUB21 in the precipitation were denatured as follows: (1.) use buffer A (50 mM Tris pH 8.0, 500 mM NaCl, 2% TritonX-100, 5 mM DTT) resuspend for ultrasonic washing for 3 times; (2.) wash once with buffer B (50 mM Tris pH 8.0, 500 mM NaCl, 5 mM DTT, 2 M urea); (3.) dissolve the inclusion body with buffer C (50 mM Tris pH 8.0, 500 mM NaCl, 5 mM DTT, 8 M urea), centrifuge the supernatant; (4.) dilute the supernatant with buffer D (50 mm Tris, 50 mm NaCl, 500 mM l- arginine, 3 mM GSSG, 1 mM GSH, 1 mM DTT, pH 8.0) were diluted and renatured for 48 h; (5.) after dilution and renaturation, dialysis to 20 mM Tris, 50 mM NaCl pH = 8.0 for 16 h, and at the end of dialysis and centrifugation, take and concentrate supernatant. After concentration, the supernatant protein was subjected to 10% SDS-PAGE. Coomassie Blue Staining and Western blot showed His-GmPUB21 successfully obtained (Figure S2A,B).

### 4.5. In Vitro Ubiquitination Analysis

According to Cho et al. [65], we have performed the in vitro self-ubiquitination assay experiment. The ubiquitination reaction mixtures (30 µL) are composed of 100 ng human E1 (E-304, Boston Biochem, Waltham, MA, USA), 100 ng human E2 (E2-627, Boston Biochem, Waltham, MA, USA), 2 µg ubiquitin (U-100AT, Boston Biochem, R & D Systems, Boston, MA, USA) and 2 µg purified E3 His-GmPUB21 was incubated in reaction buffer [50 mM Tris-HCl (pH 7.5), 2.5 mM MgCl_2_, 2 mM DTT, 4 mM ATP]. The reactions were ended by adding sample buffer and heating at 95 °C for 10 min, following 2 h of incubation at 30 °C with agitation in an Eppendorf Thermomixer. The results were detected with the support of Western blot following the standard method [66] using an anti-ubiquitin antibody (A-5; sc-166553, Santa Cruz Biotechnology, Santa Cruz, CA, USA). Tanon-5200 Chemiluminescent Imaging System (Tanon Science and Technology, Nanjing, China) was used to view the Images.

The incubation treatment of protein His-GmPUB21 followed these steps: the crude extraction was obtained from the fresh soybean leaves with buffer E (40 mM Tris-HCl, pH 7.4, 5 mM MgCl_2_, 5 mM ATP, 1 mM PMSF, 1X Protease Cocktail); the purified protein His-GmPUB21 was incubated with His-Select^®^ Nickel magnetic agarose beads (H9914, Sigma) at 4 °C for 2 h to make the protein bind with the magnetic beads; the supernatant was separated with Magnetic Separation Rack and discarded, and then beads was washed with buffer F (Tris-HCl pH 7.4, 1 mM PMSF) 3-4 times and incubated with crude extraction at 25 °C for 1 hour; then, similarly, the crude extraction was discarded and beads were washed with buffer B, 3–4 times. The pre-incubated protein His-GmPUB21 was used for verified in vitro ubiquitin activity using the same method as above.

### 4.6. Subcellular Localization of GmPUB21

The full-length *GmPUB21* coding sequence was cloned into KpnI and BamH I sites of the *pBinGFP4* vector, and it contains a *CaMV 35S: GFP*. The resultant plasmids were inserted into *Agrobacterium* tumefaciens strain EHA 105 and infiltrated into *N. benthamiana* leaves for transient assays. The GFP fluorescence was visualized by confocal laser scanning microscopy (Zeiss LSM 780, Carl Zeiss, Jena, Germany). GFP (green) and chloroplast (red) were excited at 488 nm, 500–550 nm was used to detect the fluorescence of GFP (green) and 560–650 nm were used to detect chloroplast; RFP was excited at 561 nm, and the emission was detected at 580–630 nm.

### 4.7. Generation of GmPUB21 Overexpressing Tobacco Plants

*N. benthamiana* seeds were sterilized for 7 min in 10% H_2_O_2_, washed three times using sterile water, soaked for 2 min in 75% ethanol, and washed with sterile water five times. Then, the seeds were placed on 1/2 Murashige and Skoog medium with the supplement of 2% sucrose in a plant culture container and cultured in the chamber at 23 ± 2 °C with 50 µM photons/s/m^2^ under a relative humidity of 60 ± 5% and photoperiod conditions (16-h light/8-h dark). The recombinant expression vector *35S: GmPUB21: EGFP* described above was introduced into *N. benthamiana* using *Agrobacterium tumefaciens* strain EHA105 via leaf disc transformation [67]. Transformants were chosen on MS medium with 50 mg/L Kanamycin, and PCR analysis was used to verify the presence of the transgene in T_0_, T_1_, and T_2_. The *GmPUB21* expression in T_2_ lines was analyzed by real-time PCR analysis. The lines with a higher expression of *GmPUB21* were selected, and harvested seeds of those lines were considered T_3_ seeds were used for further experiments.

### 4.8. Determination of GmPUB21 Overexpressing Tobacco Plants Seed Germination under Osmotic Stress and ABA Treatment

For the germination experiment, transgenic and WT tobacco seeds were kept on a 1/2 MS solid medium containing mannitol/NaCl (0, 100, 200, 300 mM) for 12 days and ABA (0, 0.5, 1.0, 1.5 µM) for 8 days [68,69] in a growth chamber at 25 ± 2 °C with a relative humidity of 65 ± 5% at 16 h light/8 h dark conditions. Once the radicles had entirely penetrated the seed coat, seeds were noted as germinated, and the germination rate was calculated as follows, germination rate = germinated seeds/total seeds ×100 [18]. The experiments were carried out three times.

### 4.9. Determination of GmPUB21 Overexpressing Tobacco Plant’s Response to Drought and Salt Stress

Transgenic and WT tobacco seeds were sown in pots (X1020, 54 × 28 × 7.5 cm, 15 wells) and put into the tray (54 × 28 × 6.5 cm) with the ratio 2:1 of vermiculite:nutrient soil. Seedlings were grown in the greenhouse at 25 ± 2 °C under photoperiod conditions (16 h light/8 h dark) with a relative humidity of 60 ± 5% and artificial (LED) was used to provide the light source with illumination of (50 ± 5 µM photons/s/m^2^). After 3 weeks, 1.5 L water (control) and 250 mM NaCl were added into the tray to be subjected salinity stress every time and once every 3 days for 3 weeks [70]. Similarly, we withheld water from the tobacco seedlings to cause drought stress for 12 days and then re-watered the plants for 5 days to measure the survival rate. Following the rewatering treatment, plants were considered survivors if they had green and healthy new leaves. The survival rate was measured by dividing the number of plants that survived over the total number of plants treated. After treatment, phenotypes were recorded. All the experiments were repeated three times.

### 4.10. DAB and NBT Staining

Transgenic and WT tobacco seeds were sown in pots (hp005, 5.3 × 3.5 × 5.8 cm) and grown in chamber at 25 ± 2 °C and relative humidity of 65 ± 5% at 16 h light/8 h dark conditions with the illumination of (50 ± 5 µM photons/s/m^2^), and then subjected to drought (withholding water) and salinity (250 mM NaCl) stress for 3 days [71]. DAB and NBT staining were done following the method of Negi et al. [72]. The leaves were floated in 50 mM sodium phosphate (pH 7.5) with NBT (0.2%) for O_2_^−^ detection, and the dark blue insoluble formazan product generated through the interaction of NBT with O_2_^−^ was observed and pictures were taken. DAB solution (1 mg mL^−^^1^, pH 3.8) was used to soak the leaves overnight to detect H_2_O_2_. After that, chlorophyll was detached by boiling for 10 min in ethanol, with the reddish-brown color indicating the H_2_O_2_ content was photographed.

### 4.11. Guard Cell Stomatal Aperture Observations and Measurement of Transpiration Rates

Transgenic and WT seeds were sown in pots (hp005) and transferred to the growth chamber at 25 ± 2 °C with relative humidity of 65 ± 5% at 16 h light/8 h dark conditions. To observe the changes in the guard cells, the leaf discs (1 cm in diameter) of mature rosette leaves were dissected and dipped in a stomatal opening solution (10 mM KCl, 100 mM CaCl2, and 10 mM MES, pH 6.1) for 2 h at 22 °C under white light at 450–465 nm and the illumination is (50 ± 5 µM photons/s/m^2^) [73].

Further, the leaf discs were subjected to osmotic stress including 300 mM mannitol and 250 mM NaCl for 3 h, in addition, treated with ABA (50 µM) for 2 h [74]. A high-luminosity true-color LED microscope was used to picture epidermal cells that had been scraped and placed on slides (BX53, Olympus, Tokyo, Japan). The stomatal aperture was determined in over 50 guard cells from each sample. For stomatal density, the samples were taken at a similar location in each plant, photographed under a 20× microscope and the number of stomata was counted. Five plants of each lines including wild type, were selected to participate in the survey, and the average number of stomata was calculated as a measure of stomatal density.

Water loss was calculated as described by Liu et al. [75] and Negi et al. [72]. Leaves were removed and weighed for the fresh weight from 4-week-old transgenic and WT plants. To determine the dry weight of the separated aerial pieces, they were dried at 80 °C for 2 days. To exclude variability owing to plant size or dry weight, the water content was normalized as a percentage proportional to the initial water content of the aerial parts of the plant; computed as [(W_F_ − W_T_)/(W_F_ − W_D_)] × 100, W_D_ was the dry weight, and W_F_ and W_T_ were the fresh weights for any given time interval and the original fresh weight, correspondingly [75]. These assessments were carried out in the laboratory at 25 °C and 50 ± 5% relative humidity under the illumination of 50 µM photons/s/m^2^.

### 4.12. Knock-Down of GmPUB21 in Soybean

BPMV has been proven to be effective in overexpressing and silencing endogenous genes in soybean by virus-induced gene silencing (VIGS). The procedure for constructing BPMV silencing and overexpressing vectors and inoculating plasmids was as follows by Zhang et al. [76]. In this study, to construct a gene-silencing vector of *GmPUB21*, 336 bp fragment from *GmPUB21* was amplified and inserted into pBPMV-IA-V2 (RNA2). The vectors were linked by homologous recombination (ClonExpress^®^ II One Step Cloning Kit, C112, Vazyme), and then we extracted the plasmids of RNA1 and recombinant RNA2. Further, the plasmids of recombinant RNA1 and RNA2 were mixed in equal proportion at the same concentration and used to inoculate the Soybean cv Nannong 1138-2 (virus-susceptible variety) at the VC stage. The BPMV empty vector (V) was used as the control. When the first trifoliate leaves emerged with BPMV symptoms, the target gene expression was confirmed using quantitative real-time PCR analysis. Then, BPMV symptomatic leaves were collected and frozen at −80 °C for further study. The primers used for constructing the overexpressing and silencing experiments are listed in Table S1.

### 4.13. Determination of GmPUB21 Knock-Down Plants to Drought and Salinity Stress

Soybean cultivar ‘Willimas 82’ was sown in the pots (A190) with the ratio 2:1 of vermiculite: nutrient soil and grown in the greenhouse at 25 ± 2 °C with a relative humidity of 60 ± 5% under photoperiod conditions 16 h light/8 h dark, and an illumination of 100 ± 5 µM photons/s/m^2^. At the vegetative cotyledon (VC) stage, the first true leaves of soybean plants were inoculated with fresh leaf homogenate containing recombinant BPMV (*SilGmPUB21)* and BPMV empty vector (control), respectively. After 7 days, the first compound leaves exhibited the BPMV symptoms. Before drought treatment, seedlings were grown with regular irrigation. Drought stress was imposed by withholding water for 7 days, Then, the plants were re-watered for 7 days to calculate the survival rate. For salt stress, plants were irrigated with 200 mL of 200 mM NaCl per pot in 3-day intervals for 10 days. After treatment, plants were considered survivors if they had green and healthy new leaves. The survival rate was measured by dividing the number of plants that survived over the total number of plants treated. All the experiments were carried out three times.

### 4.14. Statistical Analysis

All the measurements were replicated three times biologically. Analysis of variance was performed using SPSS Statistics 20 (SPSS Inc., Chicago, IL, USA). The student’s *t*-test was employed to assess variances among plant lines or treatments at *p* = 0.05.

## 5. Conclusions

In conclusion, U-box E3 ubiquitin ligases *GmPUB21* was identified and functionally characterized. Overexpression of *GmPUB21* increased the sensitivity to drought, salinity, and ABA treatments, while silencing the *GmPUB21* in soybean plants enhanced the tolerance to drought and salinity. Furthermore, GmPUB21 improved the stomatal density and accelerated the loss of water, decreasing the plant’s tolerance to drought and salt stress, and this response was also linked to ABA signaling. This finding expanded our knowledge of *GmPUB21* role in drought and salt stress responses.

## Data Availability

Not applicable.

## Supplementary Materials

The following supporting information can be downloaded at: https://www.mdpi.com/article/10.3390/ijms23136893/s1.

## Abbreviations

PEG	Polyethylene glycol
ABA	Abscisic acid
BiFC	Bimolecular fluorescent complimentary assay
CDS	Coding sequences
OE	overexpression
dpt	days post-treatment
Ub	Ubiquitin
E1	ubiquitin-activating enzyme
E2	ubiquitin-conjugating enzyme
E3	ubiquitin ligase
PUB	plant U-box
*N. benthamiana*	*Nicotiana benthamiana*
ROS	reactive oxygen species
VIGS	virus-induced gene silencing
BPMV	bean pod mottle virus
