# Supplementary material for: The E3 Ligase GmPUB21 Negatively Regulates Drought and Salinity Stress Response in Soybean
Source: Int J Mol Sci. 2022 Jun 21;23(13):6893. doi: 10.3390/ijms23136893 (PMC9266294; doi:10.3390/ijms23136893)
Supplement: Supplementary file 1 [file ijms-23-06893-s001.zip › ijms-1768460-supplementary.pdf]

The E3 ligase GmPUB21 negatively regulates drought and salinity stress in soybean

Yunhua Yang [1,†], Adhimoolam Karthikeyan [2, †], Jinlong Yin [1], Tongtong Jin [1], Rui Ren [3], Fei Fang [1], Han Cai [1], Mengzhuo Liu [1], Dagang Wang [1], Haijian Zhi[1*] and [1]Kai Li [1*]

[1]National Center for Soybean Improvement, National Key Laboratory for Crop Genetics and Germplasm Enhancement, Key Laboratory of Biology and Genetic Improvement of Soybean-Ministry of Agriculture, Nanjing Agricultural University, Nanjing 210095, China

[2]Subtropical Horticulture Research Institute, Jeju National University, Jeju 63243, South Korea

[3]College of Agronomy & Center for Crop Genome Engineering, Henan Agricultural University, Zhengzhou 450046, Henan, China

[†]These authors contributed equally to this work

*Correspondence: Haijian Zhi (zhj@njau.edu.cn); Kai Li (kail@njau.edu.cn)

Supporting Information

Table S1. Details of primers used in this study.

S.No	Primer ID	Forward	Reverse	Purpose
1	GmPUB21	TACAATTCTTCTAAGCTCTACCCA	TGTTTCTACAGTATGATCAACCTATAC	Cloning
2	qPCR-GmPUB21	TTATCCACAAGGCAAAACGCTG	TGGAACCACTCCCAACAGGCT	qPCR
3	Tubulin	GGAGTTCACAGAGGCAGAG	CACTTACGCATCACATAGCA	qPCR
4	pBFP4	CAAGCAATCAAGCATTCTAC	CGGACACGCTGAACTTGTGG	localization
5	PGDC-CX2	CGGATCATCTAGAACTAGTGGATCC	TGATATCGAATTCCTGCAGCCCGGG	localization
6	pBinGFP-GmPUB21	TTTACGAACGATAGGGTACCATGGTTTTGTCATGGACAA	TCACCATGGATCCGTCGACGAAGGGATTCTTCAAATGC	Localization/ Transgenic
7	T7	TAATACGACTCACTATA	GCTAGTTATTGCTCAGCGG	Prokaryotic expression
8	His-GmPUB21	CATGCCATGGTTTTGTCATGGACAAAAGGGAGA	CATGCTCGAGTTAGAAGGGATTCTTCAAATGCTT	Prokaryotic expression
9	BPMV-R2-C2	TGACATTCTCCTGGGAATTTCCC	CACACTTCACACATCATTACGAC	BPMV assay
10	silGmPUB21	GGAATCCTCTGCATGAGGATCCCTCAATTAACAACCCACGC	TCTCGAGGCCTGGAGTCGACGTCACCAAAACGGAGTTGAC	BPMV assay

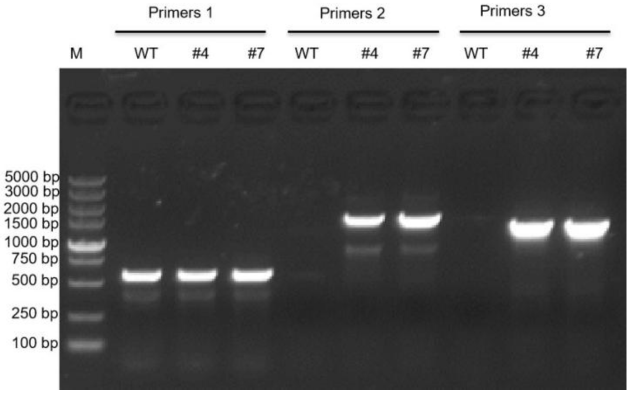

Figure S1 Transcript levels of the *GmPUB21* in wild-type and transgenic lines (#4 and #7) were analysed by RT-PCR. *GmPUB21* transcripts were present in the transgenic lines, but not in the wild-type plants. The tobacco tubulin was used as an internal reference control. Three biological experiments were done and similar results were obtained. Note; M: DL5000, WT: wild type, # 4(*OE-GmPUB21#4*), #7 (*OE-GmPUB21#7*). Primer 1: *Nt-Tubulin*; Primer 2: *pBFP4F/pBFP4R*, Primer 3: *qGmPUB21-F/pBFP4R*.

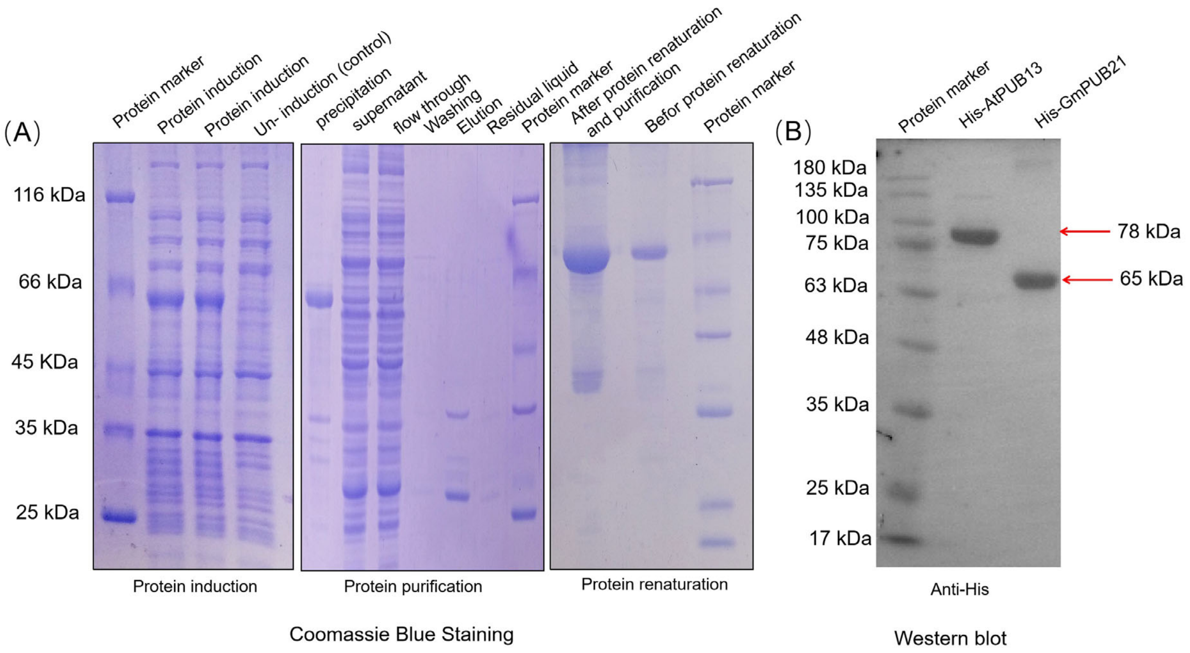

Figure S2. Prokaryotic expression and purification of His-GmPUB21. (A) His-GmPUB21 protein induction, purification and renaturation were analyzed by Coomassie Blue stain. (B) Western blot was used to detect protein His-GmPUB21 after renaturation. The results showed that the protein size of fusion protein His-GmPUB21 was 65 kDa, and His-AtPUB13 was 78 kDa.

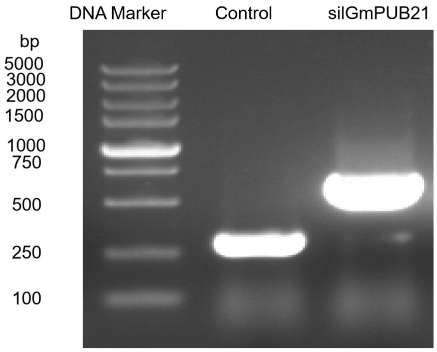

Figure S3. PCR analysis of *GmPUB21* expression in soybean plants after inoculation with BPMV empty vector (V) and BPMV based gene construct *SilGmPUB21.* The primary leaves of the soybean cv Williams 82 infected with BPMV based gene constructs and empty vector (V), *GmPUB21* expression was analysed following the emergence of BPMV symptoms in the first trifoliate leaves. *Sil-GmPUB21* and empty vector (V) infection in soybean plants confirmed by PCR.
